# Effect of species, pretreatments, and drying methods on the functional and pasting properties of high‐quality yam flour

**DOI:** 10.1002/fsn3.260

**Published:** 2015-07-20

**Authors:** Bashirat A. Wahab, Abdul‐Rasaq A. Adebowale, Silifat A. Sanni, Olajide P. Sobukola, Adewale O. Obadina, Olatundun E. Kajihausa, Mojisola O. Adegunwa, Lateef O. Sanni, Keith Tomlins

**Affiliations:** ^1^ Department of Food Science and TechnologyFederal University of AgricultureAbeokutaNigeria; ^2^ Department of Nutrition and DieteticsFederal University of AgricultureAbeokutaNigeria; ^3^ Department of Hospitality and TourismFederal University of AgricultureAbeokutaNigeria; ^4^ Natural Resources InstituteUniversity of GreenwichKentU.K

**Keywords:** *Dioscorea* sp., drying, functional properties, pretreatment, yam

## Abstract

The study investigated the functional properties of HQYF (high‐quality yam flour) from tubers of four *dioscorea species*. The tubers were processed into HQYF using two pretreatments (potassium metabisulphite: 0.28%, 15 min; blanching: 70°C, 15 min) and drying methods (cabinet: 60°C, 48 h; sun drying: 3 days). Significant differences (*P* < 0.05) were observed in pasting characteristics of flours among the four species. The drying method significantly affected only the peak viscosity. The interactive effect of species, pretreatment, and drying methods on the functional properties was significant (*P* < 0.05) except for emulsification capacity, angle of repose, and least gelation concentration. The significant variation observed in most of the functional properties of the HQYF could contribute significantly to breeding programs of the yam species for diverse food applications. The pastes of flour from *Dioscorea dumetorum* pretreated with potassium metabisulphite and dried under a cabinet dryer were stable compared to other samples, hence will have better applications in products requiring lower retrogradation during freeze/thaw cycles.

## Introduction

Yam (*Dioscorea* sp.) is an important source of carbohydrate for many people of the sub‐Saharan Africa, especially in the yam zone of West Africa (Akissoe et al. 2003). It is one of the important crops in the farming systems of Nigeria with more than 2.8 million hectares of land under cultivation annually (IITA, 2002). It contributes significantly to dietary calories per capita daily and serves as an important source of income to the people (Olaoye and Oyewole 2012). There are many varieties of yam species widespread throughout the humid tropics, but the edible yams are derived mainly from about 10. The most economically important species are white yam (*Dioscorea rotundata poir*), yellow yam (*Dioscorea cayenesis*), water yam (*Dioscorea alata*), and bitter yam (*Dioscorea dumetorum*) (Kay 1987).

Fresh yams are difficult to store and are subject to deterioration during storage (Afoakwa and Sefa‐Dedeh 2001). Olayemi et al. (2012) reported postharvest losses of yam in Nigeria to be about 37% which underscores the need for processing this staple food crop into product(s) of longer shelf life such as flour. Yam tubers have been used as a traditional food in home with little industrial use; however the traditional uses are diverse and the crop has more utilization potentials. The main shelf‐stable product of yam is the traditional yam flour (*elubo*) with little or no industrial applications. High‐quality yam flour is a novel product of yam which is produced from wholesome fresh tubers. It is odorless, crystal white, and free from foreign or extraneous material. It could find wide applications in the baking and confectionery industries. The HQYF (High‐quality yam flour) can be easily stored for a longer period (12–18 months) if the flour has low moisture.

The slow progress in upgrading the traditional food processing and preservation techniques in West Africa contributes to food and nutrition insecurity in the subregion (Aworh 2008). In recent years, much attention has been drawn to the quality of dehydrated food products. Different drying methods have effect on the biochemical and functional properties of dehydrated products (Ogunlakin et al. 2012). Hot water blanching and sulphiting are forms of pretreatment food products are usually subjected to prior to drying in order to prevent oxidative browning. Moreno‐Perez et al. (1996) reported that blanching could be used to inactivate enzymes that may lead to quality degradation and to improve the acceptability of the final product (Babajide et al. 2006). The objective of this study was to determine the effect of species, pretreatment, and drying methods on the functional properties of high‐quality yam flour.

## Materials and Methods

### Raw material sourcing

Four species (*D. rotundata*,* D. alata*,* D. cayenesis*, and *D. dumetorum*) of freshly harvested wholesome yam tubers were obtained from local markets in Abeokuta Ogun State, Nigeria. The yams were planted between December 2012 and January 2013. No fertilizer or herbicides were applied, and hand weeding was done when necessary. The yams were harvested at 100% senescence in January 2014. Harvesting was done manually with cutlass and hoe.

### Production of high‐quality yam flour

Each species of the yam tuber was washed and peeled. The peeled samples were sliced with a stainless steel vegetable slicer into 1 mm pieces and washed in potable water. The yam slices were divided into two equal portions; a portion was blanched in water bath maintained at 70°C as the first pretreatment and other portion was sulphited by immersing them in 0.28% potassium metabisulphite (second pretreatment) solution to prevent oxidative browning. Each pretreatment was done for 15 min. For cabinet drying, each pretreated yam slice was dried at 60°C for 24 h. For sun drying, each pretreated yam slice was dried on black polythene nylon for 2 days. The dried samples were ground separately with laboratory hammer mill and sieved through a 250 *μ*m mesh screen to obtain unfermented yam flour. The yam flour samples were kept separately in airtight plastic container to prevent moisture absorption and stored at room temperature until used for further analysis.

### Determination of functional properties

#### Water absorption index

Water Absorption Index (WAI) was determined according to the method described by Anderson et al. (1969).

#### Bulk density

The bulk density of the sample was determined using the method described by Akpapunam and Markakis (1981).

#### Oil absorption capacity

This was determined by the method of Sosulski (1962) as described by Nwosu et al. (2010).

#### Least gelation concentration

The least gelation concentration was determined using the method described by Adeleke and Odedeji (2010).

#### Foaming capacity

The method used by Nwosu et al. (2010) was used with a slight modification.

#### Dispersibility

This was determined by the method described by Kulkarni et al. (1991).

#### Wettability

The wetting time as described by Nwosu et al. (2010) was determined.

#### Emulsification capacity

The procedure of Adeleke and Odedeji (2010) was adopted.

#### Angle of repose

This was determined using the method described by Olorunsola et al. (2012), 20 g of flour was poured inside a funnel of orifice diameter 0.8 cm, clamped at the height of 10 cm. It was then allowed to flow freely. The height of the heap ‘h’ and the diameter ‘D’ were measured. The angle of repose, *θ*, was calculated using the equation:

$$\varphi ={\text{Tan}}^{-1}\left(2h/D\right)$$

### Pasting characteristics

The pasting characteristics were determined with a RVA (Rapid Visco Analyser) (Model: RVA‐TecMaster, Perten, Sweden). Three grams of flour was mixed in 25 mL of water in a sample canister. The sample was thoroughly mixed and fitted into the RVA. With the use of the 12‐min profile, the slurry was heated from 50°C to 95°C with a holding time of 2 min followed by cooling to 50°C with another 2‐min holding time. Both heating and cooling were at a constant rate of 11.25°C/min with constant shear at 160 rpm. The corresponding values for peak viscosity, trough, breakdown, final viscosity, setback, peak time, and pasting temperature from the pasting profile were read on a computer connected to the RVA.

## Results and Discussion

### Functional properties of HQYF

Table 1 shows the moisture content of HQYF samples. The values ranged from 7.50% to 8.20%. Yam specie as well as the interactive effect of species, pretreatments, and drying methods had no significant (*P* > 0.05) effect on the moisture content of the HQYF samples. The moisture levels obtained in this study were within the acceptable limit of not more than 10% for long‐term storage of flour (Polycarp et al. 2012). The moisture content of the unfermented yam flour was slightly different from the 5.26–7.57% reported by Udensi et al. (2008) for traditional water yam flour. The moisture content is an index of perishability and storability of food materials, so the amounts of moisture detected in these flour samples indicate that species with low moisture content would be suitable for prolonged flour storage and more efficient for industrial processing.

**Table 1:** Final moisture content (MC) of yam after drying

Species	Pretreatments	Drying methods	Moisture content
*D. rotundata*	Blanching	Cabinet	7.70 ± 0.14
		Sun	7.90 ± 0.14
	Potassium	Cabinet	7.90 ± 0.14
		Sun	7.80 ± 0.57
*D. dumetorum*	Blanching	Cabinet	7.90 ± 0.14
		Sun	8.00 ± 0.00
	Potassium	Cabinet	7.90 ± 0.14
		Sun	7.50 ± 0.14
*D. alata*	Blanching	Cabinet	7.80 ± 0.28
		Sun	7.80 ± 0.00
	Potassium	Cabinet	8.00 ± 0.28
		Sun	7.90 ± 0.14
*D. cayenensis*	Blanching	Cabinet	8.10 ± 0.14
		Sun	8.10 ± 0.14
	Potassium	Cabinet	8.20 ± 0.00
		Sun	7.90 ± 0.42
Range			7.50 ± 0.14–8.20 ± 0.00
Mean			7.90
SD			0.17
CV			2.12
SE			0.04
p of specie (S)			***
p of pretreatment (P)			***
p of drying method (D)			ns
p of S x P			ns
p of S x D			ns
p of P x D			ns
p of S x P x D			ns

***significant at *P* < 0.05. ns not significant at *P* > 0.05.

Table 2 shows the result of functional properties of the HQYF samples. Specie significantly (*P* < 0.05) affected the functional properties of HQYF samples. The interactive effect of species, pretreatments, and drying methods on the functional properties was significant (*P* < 0.05) except on emulsification capacity, angle of repose, and least gelation capacity. HQYF from *D. dumetorum* exhibits higher water absorption index (2.86 g/g) than other varieties as shown in Table 2. Water absorption characteristics represent the ability of a product to associate with water under conditions where the water is limited (Singh 2001). High oil absorption capacity is desired in retention of flavor, improvement of palatability, extension of shelf life of bakery or meat products, meat extenders, doughnuts, pan cakes, baked goods, and soup mixes, whereas low water absorption capacity is a desirable trait in foods such as sausage, custards, and dough because these are supposed to imbibe water without dissolution of protein thereby attaining body thickening and viscosity (Seena and Sridhar 2005). Therefore, the flour from *D*. *alata* and *D*. *dumetorum* pretreated with potassium metabisulphite and dried using cabinet dryer, with the highest value of oil absorption capacity and water absorption index, respectively, can be utilized for the production of such products mentioned above.

**Table 2:** Functional properties of high‐quality yam flour samples as affected by species, pretreatments, and drying methods

Species	Pretreatments	Drying methods	Dispersibility (%)	Bulk density (g/cm^3^)	WAI	Foaming Capacity	Wettability (secs)	Emulsification	OAC	Angle of Repose (deg)	LGC (%w/v)
*D. rotundata*	Blanching	Cabinet	72.17	0.88	2.02	19.93	185.33	60.45	7.72	44.17	3.00
		Sun	69.17	0.86	2.28	20.20	75.67	64.61	7.71	42.53	5.00
	Potassium	Cabinet	70.50	0.83	1.90	23.66	210.67	61.49	7.79	43.34	5.00
		Sun	68.17	0.88	2.40	22.86	104.67	66.70	7.78	40.83	4.00
*D. dumetorum*	Blanching	Cabinet	51.17	0.73	2.14	13.13	190.00	60.41	7.63	47.34	2.00
		Sun	51.83	0.72	2.26	13.53	185.00	57.29	7.62	47.07	2.00
	Potassium	Cabinet	27.83	0.77	2.86	12.78	211.00	59.25	7.76	47.41	3.00
		Sun	28.50	0.70	2.63	10.98	190.00	62.23	7.58	45.24	2.00
*D. alata*	Blanching	Cabinet	62.83	0.88	2.32	5.05	140.00	57.07	7.73	41.62	3.00
		Sun	65.67	0.91	2.49	6.79	151.33	58.33	7.81	43.57	4.00
	Potassium	Cabinet	57.67	0.91	1.95	12.90	138.33	56.51	7.96	42.93	3.00
		Sun	65.50	0.86	2.59	9.88	134.00	59.23	7.81	48.14	4.00
*D. cayenesis*	Blanching	Cabinet	52.83	0.91	2.59	13.33	125.67	57.64	7.69	40.90	4.00
		Sun	61.00	0.91	2.55	9.81	58.67	57.07	7.56	42.57	4.00
	Potassium	Cabinet	67.50	0.81	2.19	28.72	142.33	55.83	7.66	43.66	5.00
		Sun	69.17	0.86	2.12	31.11	124.67	58.53	7.53	42.08	4.00
Range			27.83–72.17	0.70–0.91	1.90–2.86	5.05–31.11	58.67–211.00	55.83–66.70	7.53–7.96	40.83–48.14	2.00–5.00
Mean			58.84	0.84	2.33	15.92	147.96	59.54	7.73	43.96	3.56
SD			13.73	0.07	0.27	7.64	45.25	3.01	0.12	2.39	1.03
SE			3.43	0.02	0.07	1.91	11.31	0.75	0.03	0.60	0.26
p of specie (S)			***	***	***	***	***	***	***	***	***
p of pretreatment (P)			***	***	ns	***	***	ns	ns	ns	ns
p of drying method (D)			***	ns	***	ns	***	***	ns	ns	ns
p of S x P			***	***	***	***	***	ns	***	ns	ns
p of S x D			***	ns	***	ns	***	ns	***	***	ns
p of P x D			ns	ns	***	ns	***	ns	***	ns	ns
p of S x P x D			***	***	***	***	***	ns	***	ns	ns

BD, Bulk density; OAC, Oil Absorption Capacity; LGC, Least Gelation Concentration.

***significant at *P* < 0.05.

ns not significant at *P* > 0.05.

The least gelling concentration obtained for all the flours was lower than the values reported for different *D.alata* varieties (30–50%w/v) by Udensi et al. (2008), cassava flour (22%w/v) by Udensi et al. (2005), and cocoyam flour (6.0–8.0%) by Ogunlakin et al. (2012). The variation observed in this property of the yam flours could be due to the relative ratios of different constituents like proteins, carbohydrates, and lipids. Different flour has been reported to posses different least gelation concentration (Sathe et al. 1982).

The bulk density of the flour was higher than 0.64–0.76 g/cm^3^ reported for varieties of water yam flour by Udensi et al. (2008). The values obtained for bulk density are also comparable to that obtained for sweet potato flour (0.7453 g/mL) used as thickener or as a base in foods like yoghurt (USDA, 2009). This implies that yam flours could find use as a thickener in the food industries to give body and mouthfeel to food products. The high volume per gram of flour material is important in relation to its packaging. It is desirable to have high bulk density in that it offers greater packaging advantage, as greater quantity may be packed within a constant volume (Fagbemi 1999; Adepeju et al. 2011). Generally, higher bulk density is desirable for the greater ease of dispersibility and reduction in paste thickness which is an important factor in convalescent child feeding (Padmashree et al. 1987). Earlier study by Malomo et al. (2012) on dispersibility of yam flour had reported a value of 58.84% similar to the mean value obtained in this work. Dispersibility is a measure of reconstitution of flour in water. The highest value (72.17%) of dispersibility was observed on the flour samples from *D*. *rotundata* hence; it will easily reconstitute to give a fine consistency to the dough during mixing (Adebowale et al. 2008).

The values obtained for wettability ranged between 58.67 and 211 sec. It was observed that wettability increased with blanching pretreatment. It has been earlier reported that during temperature process, some of the starch in the flour may have gelatinized and in the process absorbed moisture and swelled up and consequently the flours processed from these blanched slices possess a reduced hydrophillic ability leading to reduced hydration capacity of flour and thereby increasing the values obtained for wettability as blanching temperature is doubled. This report is in agreement with the findings of Tagodoe and Nip (1994) who found that the gelatinization of taro starch increased the density of the taro flour and therefore showed a reduced ability to absorb moisture. Since lower values of wettability indicate faster reconstitution properties, blanching should be done at a lower temperature to produce flour in application which requires fast water absorption.

The emulsion capacity denotes the maximum amount of oil that can be emulsified by flour dispersion (Oluwalana and Oluwamukomi 2011). The values obtained were lower compared to values reported for a blend of sweet potato and wheat 9.68–25.40 g/mL by Adeleke and Odedeji (2010). The foam capacity of the treated water yam flour is higher than that of wheat and sweet potato flour blend (Adeleke and Odedeji 2010). Low foam capacity was observed in the flour from *D*. *alata* specie pretreated with hot water blanching and dried using the two drying methods. These flours could be desirable in food processes where excessive foaming is not required as it reduces loss due to foam spillage or the need for including an extra steep or antifoaming agent to check foaming.

### Pasting profile of HQYF

Tables 3 and 4 show the results of the pasting profile of HQYF from different species of yam tubers which undergo different pretreatment and drying methods. A significant difference (*P* < 0.01) was observed in the pasting characteristics of flours among the four species of yam tubers. Drying method significantly affected only the peak viscosity. The interaction between pretreatment and drying methods followed the same pattern as in variation between drying methods, while the interaction between specie, pretreatment, and drying methods was not significantly different (*P* > 0.05) except for peak and final viscosities.

**Table 3:** Pasting Properties (RVU) of high‐quality yam flour samples as affected by species, pretreatments, and drying methods

Species	Pretreatments	Drying methods	Peak	Trough	Breakdown	Final Viscosity	Setback
*D. rotundata*	Blanching	Cabinet	430.14	211.06	219.08	427.72	216.67
		Sun	462.03	183.11	278.92	329.31	146.19
	Potassium	Cabinet	506.81	149.47	357.33	359.97	210.50
		Sun	428.97	164.61	264.36	347.64	183.03
*D. dumetorum*	Blanching	Cabinet	267.14	139.25	127.89	200.58	61.33
		Sun	371.06	226.78	144.28	339.25	112.47
	Potassium	Cabinet	181.83	100.39	81.44	157.11	56.72
		Sun	186.75	94.44	92.31	160.33	65.89
*D. alata*	Blanching	Cabinet	491.14	404.86	86.28	588.67	183.81
		Sun	491.64	309.97	181.67	600.28	290.31
	Potassium	Cabinet	434.17	344.31	89.86	582.92	238.61
		Sun	496.08	285.19	210.89	606.64	321.44
*D. cayenesis*	Blanching	Cabinet	303.61	264.11	39.50	427.69	163.58
		Sun	455.14	397.50	57.64	649.58	252.08
	Potassium	Cabinet	440.75	209.92	230.83	378.89	168.97
		Sun	421.75	198.75	223.00	281.50	82.75
Range			181.83–506.81	94.44–404.86	39.50–357.33	157.11–649.58	56.72–321.44
Mean			398.1	230.2	167.8	402.4	172.1
SD			106.5	96.8	91.6	163.2	81.6
SE			26.6	24.2	22.9	40.8	20.4
p of species (S)			***	***	***	***	***
p of pretreatment (P)			ns	***	***	***	ns
p of drying method (D)			***	ns	ns	ns	ns
p of S x P			***	ns	***	***	ns
p of S x D			ns	***	ns	ns	ns
p of P x D			***	ns	ns	ns	ns
p of S x P x D			***	ns	ns	***	ns

***significant at *P* < 0.05.

ns not significant at *P* > 0.05.

**Table 4:** Peak time and pasting temperature of high‐quality yam flour samples as affected by species, pretreatments, and drying methods

Species	Pretreatments	Drying methods	Peak time (min)	Pasting temperature (°C)
*D. rotundata*	Blanching	Cabinet	4.7	80.9
		Sun	4.8	81.0
	Potassium	Cabinet	4.6	70.1
		Sun	4.9	79.5
*D. dumetorum*	Blanching	Cabinet	4.7	86.5
		Sun	4.7	85.4
	Potassium	Cabinet	4.9	88.4
		Sun	4.9	87.6
*D. alata*	Blanching	Cabinet	5.0	82.0
		Sun	4.7	81.6
	Potassium	Cabinet	5.5	81.8
		Sun	4.8	80.8
*D. cayenesis*	Blanching	Cabinet	5.6	71.1
		Sun	5.8	69.9
	Potassium	Cabinet	4.9	70.3
		Sun	4.6	71.4
Range			4.6–5.8	69.9–88.4
Mean			4.9	79.3
SD			0.4	6.6
SE			0.1	1.6
p of specie (S)			***	***
p of pretreatment (P)			ns	ns
p of drying method (D)			ns	ns
p of S x P			***	ns
p of S x D			***	ns
p of P x D			ns	ns
p of S x P x D			ns	ns

***significant at *P* < 0.05.

ns not significant at *P* > 0.05.

Pasting temperature values of yam flour are in agreement with pasting temperatures (85.89°C and 79.88°C, respectively) for *D*. *alata* and *D*. *rotundata* flours reported by Wireko‐Manu et al. (2011). Pasting temperature has been described as the temperature above the gelatinization temperature when starch granules begin to swell and it is characterized by an increase in viscosity on shearing (Adebowale et al. 2005). Pasting temperature provides an indication of the minimum temperature required to cook the flour and this has an implication for the suitability of other food (with different gelatinization temperature) in a food formula (Newport Scientific, 1998). The highest pasting temperature was recorded for flour from *D*. *dumetorum* dried using cabinet dryer with potassium metabisulphite as the method of pretreatment, and the lowest for *D*. *cayenesis* sun dried with blanching as the method of pretreatment. The high pasting temperature of *D*. *dumetorum* flour indicates the presence in this flour, of starch that is highly resistance to swelling and rupturing. Defloor et al. (1994) reported that attaining gelatinization at a lower temperature led to improved bread‐making quality. Therefore, blanched and sun‐dried flour from *D*. *cayenesis* could be used for bread baking.

The peak viscosity is the maximum viscosity attainable during the heating cycle. The highest value was observed in cabinet‐dried *D*. *rotundata* flour that was pretreated with potassium metabisulphite, and the lowest for *D*. *dumetorum* which had undergone the same treatment. The peak viscosity indicates the water binding capacity of the starch. It is often correlated with the final product quality and also provides an indication of the viscous load likely to be encountered during mixing (Maziya‐Dixon et al. 2007). The peak viscosity relates with the product quality, hence a significant difference observed among the species studied may influence their performance in product development. The time taken to attain peak viscosity was similar to that of *D*. *alata* and *D*. *rotundata* (4.73–7.00 min) reported by Wireko‐Manu et al. (2011). The peak time is a measure of the cooking time (Adebowale et al. 2005).

As part of the pasting characteristics studied, the flour sample subjected to RVA was heated to 95°C and held at that temperature for a couple of minutes under mechanical shear stress. As a result of starch granule disruption and the leaching out of amylose into the solution, under mechanical shear stress, viscosity decreases. The period provides the minimum viscosity value in the constant temperature pasting profile known as trough. Trough is considered as a measure of the breakdown of hot starch paste. The ability of a paste to withstand the heating and shear stress is an important factor for most food processing operations and is also a factor in describing the quality of starch gel (Madsen and Christensen 1996). High paste stability is a requirement for industrial users of starch (Bainbridge et al. 1996). This is because drastic changes in paste during and after processing could lead to undesirable textural changes. Generally, the *D*. *alata* flour had higher trough, which indicates greater ability to withstand shear at high temperatures and higher cooked paste stability (Rasper 1969; Farhat et al. 1999). Starch with a low trough value would have greater need for cross‐linking than one with a high value (Oduro et al. 2000). *D. alata* flour could therefore be targeted for industrial uses because of its hot paste stability. The lowest breakdown (measure of the ease with which the swollen granule can be disintegrated) was observed in flour from *D*. *cayenesis* that was pretreated by blanching and dried using cabinet dryer, thereby indicating the stability of the swollen granules against disintegration during cooking. The rate of starch breakdown depends on the nature of the material, the temperature and the degree of mixing, and shear applied to the mixture (Newport Scientific, 1998). The ability of a mixture to withstand heating and the shear stress that is usually encountered during processing is an important factor for many processes, especially those requiring stable paste and low retrogradation/syneresis (Sanni et al. 2008).

The setback viscosity which is an index of the retrogradation of linear starch molecules during cooling was lower than that (86.52–210.94 RVU) reported by Jimoh et al. (2009) for yam flours. Setback has been correlated with the texture of various products. High setback is also associated with syneresis or weeping during freeze/thaw cycles (Adebowale et al. 2005), while low setback during the cooling of paste from starch or a starch‐based food indicates greater resistance to retrogradation (Sanni et al. 2004). The lowest setback value of flour from *D*. *dumetorum* indicates its lower tendency to retrograde. The smaller tendencies to retrograde are advantage in food products such as soup and sauce, which undergo loss of viscosity and precipitation as a result of retrogradation (Adebowale and Lawal 2003) and for this reason flour from *D*. *dumetorum* specie may be suitable for products like soup mixes.

The final viscosity (indicates the ability of the material to form a viscous paste) has been reported as the most commonly used parameter to determine the ability of starch‐based materials to form a viscous paste or gel after cooking and cooling as well as the resistance of the paste to shear force during stirring (Adebowale et al. 2005; Maziya‐Dixon et al. 2007).

## Conclusions

The result of this study showed that species, pretreatments, and drying methods affected the functional properties of the HQYF. The wide variation observed in functional characteristics of the flour samples serves as a database for the selection and improvement of the yam species for specific food applications to stimulate their industrial processing and utilization. The flours obtained in this study could serve better as a good binder or provider of consistency in food preparations such as semisolid beverages and the better the gelling ability of the flour. The pastes of flour from *D. dumetorum* were relatively stable and hence will have a lower tendency to undergo retrogradation during freeze/thaw cycles than flour from other species.

## Conflict of Interest

None declared.
